# Supplementary material for: Pyk2 in dorsal hippocampus plays a selective role in spatial memory and synaptic plasticity
Source: Sci Rep. 2021 Aug 11;11:16357. doi: 10.1038/s41598-021-95813-x (PMC8358019; doi:10.1038/s41598-021-95813-x)
Supplement: Supplementary file 1 — Supplementary Information. [file 41598_2021_95813_MOESM1_ESM.pdf]

Pyk2 in dorsal hippocampus plays a selective role in spatial memory and synaptic plasticity

Vincenzo Mastrolia, Omar al Massadi, Benoit de Pins, Jean-Antoine Girault

Supplementary Information

Content:

Statistical analyses: Supplementary Tables 1-5

Supplementary Table 1 Statistics for Figs. 1B, 2A, and 2B

Supplementary Table 2 Statistics for Figs. 3C and 3D

Supplementary Table 3 Statistics for Figs. 3E and 3F

Supplementary Table 4 Statistics for Figs. 4D and 4E

Supplementary Table 5 Statistics for Figs. 4F and 4G

Supplementary Figure 1

Supplementary Table 1: Statistical analyses for Figures 1B, 2A and 2B

Data were analyzed with d'Agostino-Pearson and Shapiro-Wilk normality tests.
Estimation statistics was done using https://www.estimationstats.com/ which calculated the permutation t-test with 5000 reshuffles
All tests were used as two-tailed.

Figure 1B

Figure	Variable	Group	n mice	n data points	Passed normality tests	Mean	SEM	95% CI	Statistical analysis	Comparison	DF	Test value	*P* value
1B	Time spent exploring object at new location NL (% of total object exploraton time)	WT NL	17	17	Yes	63,8	2,0	59,6 ; 67,9	One-sample t-test	with 50% chance level	16	t = 6,99	< 0,0001
		Pyk2 KO NL	17	17	Yes	52,8	2,9	46,6 ; 59,0			16	t = 0,96	0,35
		Mean difference WT and KO at NL	34	34		-10,9		-17,3 ; -4,5	Permutation t-test, 5000 reshuffles of WT and KO values				0,004

Figure 2A, B

Figure	Variable	Group	n mice	n data points	Passed normality tests	Mean	SEM	95% CI	Statistical analysis	Comparison	DF	Test value	*P* value
2A	Time spent exploring object at new location NL (% of total object exploraton time)	KO AAV GFP NL	17	17	Yes	51,9	4,4	42,7 ; 61,2	One-sample t-test	with 50% chance level	16	t = 0,45	0,66
		KO AAV-GFP/Pyk2 NL	16	16	Yes	65,8	3,7	57,8 ; 73,7			15	t = 4,21	0,0008
		Mean difference GFP and GFP/Pyk2 at NL	33	33		13,8		3,2 ; 24,8	Permutation t-test, 5000 reshuffles of GFP and GFP/Pyk2 values				0,024
2B	Time spent exploring object at new location NL (% of total object exploraton time)	Ptk2bf/f AAV-GFP NL	20	20	Yes[1]	65,8	3,1	59,3 ; 72,3	One-sample t-test	with 50% chance level	19	t = 5,11	< 0,0001
		Ptk2bf/f AAV-GFP/Cre NL	22	22	Yes	53,0	3,5	45,7 ; 60,37			21	t = 0,84	0,41
		Mean difference GFP and GFP/Cre at NL	42	42		-12,9		-21,5 ; -3,4	Permutation t-test, 5000 reshuffles of GFP and GFP/Cre values				0,009

1, Shapiro-Wilk only

Supplementary Table 2: Statistical analyses for Figures 3C and 3D

Data were analyzed with d'Agostino-Pearson and Shapiro-Wilk normality tests.

Time courses were analyzed with 2-way repeated measures ANOVA and post hoc multiple comparisons after ANOVA were done with Holm-Sidak's test.

Comparison of 4 groups were done with 2-way ANOVA (Figs. 3C, 3E, 4E, 4G).

Estimation statistics was done using https://www.estimationstats.com/ which calculated the permutation t-test with 5000 reshuffles (Figs. 3C, 3E, 4E, 4G).

All tests were used as two-tailed.

Figure	Variable	Group	n mice	n slices	Passed normality test	Mean	SEM	95% CI	Statistical analysis	Comparison	DF	Test value	*P* value
**3C**	Normalized slope (% of baseline)	WT	8	13					Two-way ANOVA	Interaction	79 ; 2054	F = 4,63	**0,0012**
		Pyk2 KO	7	15						Time	79 ; 2054	F = 62,84	**< 0,0001**
										Genotype	1 ; 26	F = 15,76	**0,0005**
**3D**	Average potentiation 50 60 min after HFS	WT	8	13	Yes	156,8	7,6	140,2 ; 173,4					
		Pyk2 KO	7	15	Yes	134,2	3,8	126,0 ; 142,3					
		Mean difference between WT and Pyk2 KO	15	28		-22,6		-39,6 ; -7,6	Permutation t-test, 5000 reshuffles of WT and KO values				**0,0068**

3C Post-hoc comparisons

	Normalized slope (% of baseline)		Number of slices		Holm-Sidak's test			
**Time (min)**	**Mean WT**	**Mean KO**	**WT**	**KO**	**t**	**DF**	**Significance**	**Adj P value**
-20	98,4	100,1	13	15	0,26	2080	ns	> 0,9999
-19	100,3	99,3	13	15	0,16	2080	ns	> 0,9999
-18	100,0	104,0	13	15	0,63	2080	ns	> 0,9999
-17	101,9	102,2	13	15	0,06	2080	ns	> 0,9999
-16	96,1	98,5	13	15	0,38	2080	ns	> 0,9999
-15	100,4	103,3	13	15	0,45	2080	ns	> 0,9999
-14	97,2	101,7	13	15	0,71	2080	ns	> 0,9999
-13	97,7	99,6	13	15	0,30	2080	ns	> 0,9999
-12	101,4	101,0	13	15	0,05	2080	ns	> 0,9999
-11	99,7	101,3	13	15	0,24	2080	ns	> 0,9999
-10	97,9	97,8	13	15	0,02	2080	ns	> 0,9999
-9	102,4	99,8	13	15	0,40	2080	ns	> 0,9999
-8	95,5	98,5	13	15	0,48	2080	ns	> 0,9999
-7	103,1	100,1	13	15	0,47	2080	ns	> 0,9999
-6	101,3	101,2	13	15	0,01	2080	ns	> 0,9999
-5	99,4	100,1	13	15	0,11	2080	ns	> 0,9999
-4	100,6	97,8	13	15	0,44	2080	ns	> 0,9999
-3	100,8	97,8	13	15	0,48	2080	ns	> 0,9999
-2	102,7	96,1	13	15	1,04	2080	ns	0,9992

	-1	103,3	99,8	13	15	0,55	2080	ns	> 0,9999
HFS	0	122,9	95,0	13	15	4,40	2080	***	0,0009
	1	149,3	117,6	13	15	4,98	2080	****	< 0,0001
	2	151,0	122,0	13	15	4,55	2080	***	0,0004
	3	152,5	127,6	13	15	3,92	2080	**	0,006
	4	158,6	124,8	13	15	5,31	2080	****	< 0,0001
	5	155,0	127,5	13	15	4,31	2080	**	0,0013
	6	158,2	127,9	13	15	4,76	2080	***	0,0002
	7	153,5	130,0	13	15	3,70	2080	*	0,0141
	8	155,1	132,1	13	15	3,62	2080	*	0,0179
	9	155,9	129,9	13	15	4,09	2080	**	0,0031
	10	155,6	129,8	13	15	4,05	2080	**	0,0036
	11	154,1	128,9	13	15	3,95	2080	**	0,0054
	12	153,3	132,6	13	15	3,25	2080	ns	0,0519
	13	150,7	132,7	13	15	2,84	2080	ns	0,1204
	14	150,3	133,0	13	15	2,71	2080	ns	0,1387
	15	151,6	133,9	13	15	2,77	2080	ns	0,132
	16	149,8	133,8	13	15	2,52	2080	ns	0,2191
	17	149,6	131,9	13	15	2,78	2080	ns	0,132
	18	149,0	130,5	13	15	2,90	2080	ns	0,1041
	19	152,6	130,8	13	15	3,43	2080	*	0,0322
	20	150,0	131,1	13	15	2,98	2080	ns	0,0929
	21	151,9	132,4	13	15	3,08	2080	ns	0,0776
	22	152,0	134,6	13	15	2,73	2080	ns	0,1379
	23	150,6	131,0	13	15	3,08	2080	ns	0,0776
	24	155,7	129,5	13	15	4,12	2080	**	0,0028
	25	152,0	131,5	13	15	3,22	2080	ns	0,0535
	26	156,4	133,7	13	15	3,58	2080	*	0,0201
	27	152,3	133,4	13	15	2,98	2080	ns	0,0929
	28	155,6	133,5	13	15	3,47	2080	*	0,0285
	29	154,3	133,4	13	15	3,29	2080	ns	0,0503
	30	156,2	133,1	13	15	3,62	2080	*	0,0179
	31	156,7	132,4	13	15	3,83	2080	**	0,0085
	32	154,9	133,3	13	15	3,39	2080	*	0,0364
	33	153,1	135,2	13	15	2,82	2080	ns	0,1243
	34	153,4	133,3	13	15	3,15	2080	ns	0,0651
	35	152,1	132,6	13	15	3,06	2080	ns	0,0776
	36	153,0	133,9	13	15	3,00	2080	ns	0,088
	37	153,8	134,9	13	15	2,98	2080	ns	0,0929
	38	153,9	131,4	13	15	3,53	2080	*	0,0239
	39	156,0	135,2	13	15	3,27	2080	ns	0,0516
	40	154,9	132,0	13	15	3,60	2080	*	0,0191
	41	155,5	134,8	13	15	3,27	2080	ns	0,0517
	42	156,4	133,3	13	15	3,63	2080	*	0,0178
	43	153,0	135,3	13	15	2,78	2080	ns	0,132
	44	154,6	134,0	13	15	3,24	2080	ns	0,0521
	45	153,3	132,1	13	15	3,34	2080	*	0,042
	46	154,1	133,4	13	15	3,26	2080	ns	0,0519

47	153,5	132,8	13	15	3,25	2080	ns	0,0519
48	158,4	131,6	13	15	4,22	2080	**	0,0018
49	154,5	134,4	13	15	3,15	2080	ns	0,0651
50	154,2	134,4	13	15	3,12	2080	ns	0,0689
51	156,0	134,4	13	15	3,40	2080	*	0,0355
52	158,1	139,2	13	15	2,96	2080	ns	0,0929
53	157,5	136,9	13	15	3,24	2080	ns	0,0521
54	158,4	133,2	13	15	3,96	2080	**	0,0054
55	156,6	131,5	13	15	3,95	2080	**	0,0054
56	156,7	134,5	13	15	3,48	2080	*	0,0284
57	154,9	135,4	13	15	3,08	2080	ns	0,0776
58	159,7	131,1	13	15	4,50	2080	***	0,0006
59	158,2	131,0	13	15	4,27	2080	**	0,0015

Supplementary Table 3: Statistical analyses for Figures 3E and 3F

Data were analyzed with d’Agostino-Pearson and Shapiro-Wilk normality tests.

Time courses were analyzed with 2-way repeated measures ANOVA and post hoc multiple comparisons after ANOVA were done with Holm-Sidak’s test.

Comparison of 4 groups were done with 2-way ANOVA (Figs. 3C, 3E, 4E, 4G).

Estimation statistics was done using https://www.estimationstats.com/ which calculated the permutation t-test with 5000 reshuffles (Figs. 3C, 3E, 4E, 4G).

All tests were used as two-tailed.

Figure	Variable	Group	n mice	n slices	Time (min)	Mean	SEM	95% CI	Statistical analysis	Comparison	DF	Test value	*P* value
3E	Normalized slope (% of baseline)	WT	5	8					Two-way ANOVA	Interaction	79 ; 948	F = 0,63	0,99
		Pyk2 KO	5	6						Time	79 ; 948	F = 17,87	**< 0,0001**
										Genotype	1 ; 12	F = 0,10	0,75
3F	Average potentiation 50 60 min after TBS	WT	5	8	Yes	136,7	8,6	116,3 ; 157,0					
		Pyk2 KO	5	6	N too small	134,6	5,7	119,9 ; 149,3					
		Mean difference between WT and Pyk2 KO	10	14		-2,1		-20,5 ; 16,8	Permutation t-test, 5000 reshuffles of WT and KO values				0,85

3E Post-hoc comparisons

	Normalized slope (% of baseline)		Number of slices		Holm-Sidak's test			
Time (min)	Mean WT	Mean KO	N WT	N KO	t	DF	Significance	Adj P value
-20	96,9	94,7	8	6	0,25	960	ns	> 0,9999
-19	98,4	100,7	8	6	0,28	960	ns	> 0,9999
-18	103,1	93,8	8	6	1,09	960	ns	> 0,9999
-17	96,9	94,4	8	6	0,29	960	ns	> 0,9999
-16	101,9	94,0	8	6	0,92	960	ns	> 0,9999
-15	102,6	100,5	8	6	0,24	960	ns	> 0,9999
-14	97,0	99,0	8	6	0,22	960	ns	> 0,9999
-13	97,9	99,0	8	6	0,13	960	ns	> 0,9999
-12	97,1	103,6	8	6	0,76	960	ns	> 0,9999
-11	103,4	96,2	8	6	0,84	960	ns	> 0,9999
-10	98,4	103,5	8	6	0,60	960	ns	> 0,9999
-9	98,2	97,7	8	6	0,06	960	ns	> 0,9999
-8	98,3	103,6	8	6	0,62	960	ns	> 0,9999
-7	99,8	103,0	8	6	0,39	960	ns	> 0,9999
-6	97,0	103,9	8	6	0,81	960	ns	> 0,9999
-5	101,8	102,8	8	6	0,12	960	ns	> 0,9999
-4	99,2	96,7	8	6	0,30	960	ns	> 0,9999
-3	103,2	109,6	8	6	0,75	960	ns	> 0,9999
-2	108,1	98,9	8	6	1,07	960	ns	> 0,9999

	-1	100,9	104,3	8	6	0,39	960	ns	> 0,9999
**TBS**	0	91,5	92,8	8	6	0,15	960	ns	> 0,9999
	1	116,4	124,3	8	6	0,93	960	ns	> 0,9999
	2	126,4	125,4	8	6	0,12	960	ns	> 0,9999
	3	128,5	123,6	8	6	0,58	960	ns	> 0,9999
	4	131,2	126,4	8	6	0,56	960	ns	> 0,9999
	5	129,6	136,4	8	6	0,79	960	ns	> 0,9999
	6	128,7	124,4	8	6	0,51	960	ns	> 0,9999
	7	124,7	127,6	8	6	0,33	960	ns	> 0,9999
	8	128,0	125,2	8	6	0,33	960	ns	> 0,9999
	9	125,8	124,3	8	6	0,18	960	ns	> 0,9999
	10	123,4	121,9	8	6	0,17	960	ns	> 0,9999
	11	121,3	121,3	8	6	0,00	960	ns	> 0,9999
	12	122,8	122,1	8	6	0,08	960	ns	> 0,9999
	13	122,1	125,0	8	6	0,34	960	ns	> 0,9999
	14	119,0	118,4	8	6	0,07	960	ns	> 0,9999
	15	126,3	123,1	8	6	0,37	960	ns	> 0,9999
	16	127,1	131,4	8	6	0,51	960	ns	> 0,9999
	17	126,5	127,1	8	6	0,06	960	ns	> 0,9999
	18	121,1	123,2	8	6	0,25	960	ns	> 0,9999
	19	121,2	130,4	8	6	1,08	960	ns	> 0,9999
	20	124,4	129,7	8	6	0,61	960	ns	> 0,9999
	21	122,3	129,1	8	6	0,79	960	ns	> 0,9999
	22	121,2	130,9	8	6	1,14	960	ns	> 0,9999
	23	120,8	131,0	8	6	1,20	960	ns	> 0,9999
	24	125,6	134,6	8	6	1,06	960	ns	> 0,9999
	25	126,0	132,9	8	6	0,80	960	ns	> 0,9999
	26	125,9	124,3	8	6	0,19	960	ns	> 0,9999
	27	122,6	132,3	8	6	1,13	960	ns	> 0,9999
	28	125,8	132,6	8	6	0,80	960	ns	> 0,9999
	29	121,0	130,3	8	6	1,09	960	ns	> 0,9999
	30	125,2	127,9	8	6	0,32	960	ns	> 0,9999
	31	125,9	132,7	8	6	0,80	960	ns	> 0,9999
	32	121,9	131,4	8	6	1,11	960	ns	> 0,9999
	33	127,3	130,7	8	6	0,40	960	ns	> 0,9999
	34	126,2	128,6	8	6	0,27	960	ns	> 0,9999
	35	125,8	132,4	8	6	0,77	960	ns	> 0,9999
	36	125,8	127,9	8	6	0,24	960	ns	> 0,9999
	37	127,1	138,4	8	6	1,32	960	ns	> 0,9999
	38	132,2	137,3	8	6	0,60	960	ns	> 0,9999
	39	127,4	132,9	8	6	0,64	960	ns	> 0,9999
	40	125,4	130,7	8	6	0,62	960	ns	> 0,9999
	41	133,2	135,0	8	6	0,21	960	ns	> 0,9999
	42	130,7	126,5	8	6	0,50	960	ns	> 0,9999
	43	130,4	133,4	8	6	0,36	960	ns	> 0,9999
	44	128,9	134,8	8	6	0,69	960	ns	> 0,9999

45	129,1	135,7	8	6	0,77	960	ns	> 0,9999
46	132,2	140,6	8	6	0,99	960	ns	> 0,9999
47	132,3	133,0	8	6	0,09	960	ns	> 0,9999
48	135,9	137,5	8	6	0,19	960	ns	> 0,9999
49	128,3	133,6	8	6	0,63	960	ns	> 0,9999
50	135,7	135,7	8	6	0,01	960	ns	> 0,9999
51	141,8	131,6	8	6	1,19	960	ns	> 0,9999
52	136,7	134,5	8	6	0,26	960	ns	> 0,9999
53	135,8	132,8	8	6	0,36	960	ns	> 0,9999
54	135,8	134,9	8	6	0,10	960	ns	> 0,9999
55	139,6	132,9	8	6	0,79	960	ns	> 0,9999
56	135,9	135,3	8	6	0,07	960	ns	> 0,9999
57	137,3	141,5	8	6	0,49	960	ns	> 0,9999
58	139,0	132,1	8	6	0,80	960	ns	> 0,9999
59	137,5	132,5	8	6	0,58	960	ns	> 0,9999

Supplementary Table 4: Statistical analyses for Figures 4D and 4E

Data were analyzed with d'Agostino-Pearson and Shapiro-Wilk normality tests.
Time courses were analyzed with 2-way repeated measures ANOVA and post hoc multiple comparisons after ANOVA were done with Holm-Sidak's test.
Comparison of 4 groups were done with 2-way ANOVA (Figs. 3C, 3E, 4E, 4G).
Estimation statistics was done using https://www.estimationstats.com/ which calculated the permutation t-test with 5000 reshuffles (Figs. 3C, 3E, 4E, 4G).
All tests were used as two-tailed.

Figure	Variable	Group	n mice	n slices	Passed normality test	Mean	SEM	95% CI	Statistical analysis	Comparison	DF	Test value	*P* value
**4D**	Normalized slope (% of baseline)	AAV-GFP	4	8					Two-way ANOVA	Interaction	79 ; 1027	F = 5,13	**< 0,0001**
		AAV-GFP/Cre	4	7						Time	79 ; 1027	F = 19,83	**< 0,0001**
										Genotype	1 ; 13	F = 12,16	**0,004**
**4E**	Average potentiation 50-60 min after HFS	AAV-GFP	4	8	Yes	158,7	9,8	135,6 ; 181,8					
		AAV-GFP/Cre	4	7	Yes[1]	111,8	10,8	87,1 ; 136,5					
		Mean difference between GFP and Cre	8	15		-46,9		-74,0 ; -22,2	Permutation t-test, 5000 reshuffles of GFP and Cre values				**0,0064**

1, Shapiro-Wilk only

4D Post-hoc comparisons

	Normalized slope (% of baseline)		Number of slices		Holm-Sidak's test			
**Time (min)**	**Mean GFP**	**Mean Cre**	**AAV-GFP**	**AAV-GFP/Cre**	**t**	**DF**	**Significance**	**Adj P value**
-20	103,4	94,1	7	8	0,87	1040	ns	> 0,9999
-19	101,5	92,3	7	8	0,86	1040	ns	> 0,9999
-18	100,5	96,3	7	8	0,39	1040	ns	> 0,9999
-17	102,3	97,2	7	8	0,47	1040	ns	> 0,9999
-16	95,9	98,6	7	8	0,25	1040	ns	> 0,9999
-15	99,2	99,9	7	8	0,06	1040	ns	> 0,9999
-14	103,7	102,0	7	8	0,17	1040	ns	> 0,9999
-13	98,8	99,7	7	8	0,09	1040	ns	> 0,9999
-12	97,1	98,7	7	8	0,15	1040	ns	> 0,9999
-11	96,1	100,6	7	8	0,42	1040	ns	> 0,9999
-10	98,2	97,4	7	8	0,08	1040	ns	> 0,9999
-9	98,8	100,0	7	8	0,12	1040	ns	> 0,9999
-8	101,9	99,5	7	8	0,23	1040	ns	> 0,9999
-7	102,9	99,1	7	8	0,36	1040	ns	> 0,9999
-6	99,4	101,3	7	8	0,18	1040	ns	> 0,9999
-5	100,2	103,3	7	8	0,29	1040	ns	> 0,9999
-4	99,5	102,5	7	8	0,27	1040	ns	> 0,9999
-3	105,0	107,5	7	8	0,24	1040	ns	> 0,9999
-2	100,9	104,6	7	8	0,35	1040	ns	> 0,9999
-1	94,8	105,6	7	8	1,02	1040	ns	0,9998

**HFS**	0	119,3	122,7	7	8	0,32	1040	ns	> 0,9999
	1	130,3	139,4	7	8	0,85	1040	ns	> 0,9999
	2	132,4	141,0	7	8	0,81	1040	ns	> 0,9999
	3	129,6	145,9	7	8	1,54	1040	ns	0,9595
	4	127,6	147,5	7	8	1,87	1040	ns	0,8098
	5	129,3	149,8	7	8	1,92	1040	ns	0,7831
	6	132,5	156,5	7	8	2,26	1040	ns	0,5079
	7	130,5	154,0	7	8	2,21	1040	ns	0,5427
	8	131,4	156,8	7	8	2,38	1040	ns	0,4087
	9	133,2	158,9	7	8	2,41	1040	ns	0,3984
	10	131,6	159,0	7	8	2,57	1040	ns	0,2807
	11	129,8	157,6	7	8	2,61	1040	ns	0,2773
	12	137,5	157,3	7	8	1,86	1040	ns	0,8098
	13	130,7	158,4	7	8	2,60	1040	ns	0,2773
	14	126,1	157,7	7	8	2,97	1040	ns	0,1179
	15	128,7	156,3	7	8	2,59	1040	ns	0,2773
	16	124,7	158,5	7	8	3,17	1040	ns	0,0654
	17	126,7	155,7	7	8	2,73	1040	ns	0,2189
	18	125,5	153,8	7	8	2,65	1040	ns	0,2543
	19	125,8	154,7	7	8	2,71	1040	ns	0,2228
	20	125,3	154,8	7	8	2,77	1040	ns	0,1998
	21	123,0	156,9	7	8	3,19	1040	ns	0,0642
	22	124,3	159,9	7	8	3,34	1040	*	0,0412
	23	118,7	156,9	7	8	3,59	1040	*	0,02
	24	122,7	156,6	7	8	3,18	1040	ns	0,0647
	25	123,9	155,1	7	8	2,93	1040	ns	0,1317
	26	117,6	157,9	7	8	3,78	1040	*	0,0106
	27	120,0	160,0	7	8	3,76	1040	*	0,0111
	28	122,6	158,1	7	8	3,34	1040	*	0,0412
	29	120,5	157,7	7	8	3,50	1040	*	0,0261
	30	123,6	159,9	7	8	3,41	1040	*	0,0348
	31	122,2	158,1	7	8	3,37	1040	*	0,0383
	32	122,3	160,1	7	8	3,55	1040	*	0,0224
	33	120,4	156,0	7	8	3,34	1040	*	0,0412
	34	121,0	155,3	7	8	3,22	1040	ns	0,0588
	35	118,0	158,8	7	8	3,83	1040	**	0,009
	36	126,6	158,8	7	8	3,03	1040	ns	0,1016
	37	119,3	156,7	7	8	3,51	1040	*	0,025
	38	115,6	156,5	7	8	3,85	1040	**	0,0085
	39	121,1	157,1	7	8	3,39	1040	*	0,0368
	40	117,3	156,4	7	8	3,67	1040	*	0,0154
	41	120,3	157,3	7	8	3,47	1040	*	0,0279
	42	116,8	156,8	7	8	3,75	1040	*	0,0112
	43	113,2	155,0	7	8	3,93	1040	**	0,0063
	44	117,0	157,2	7	8	3,78	1040	*	0,0107
	45	118,0	156,2	7	8	3,59	1040	*	0,02

46	118,5	156,9	7	8	3,61	1040	*	0,019
47	113,0	157,5	7	8	4,18	1040	**	0,0022
48	113,1	153,2	7	8	3,76	1040	*	0,0111
49	111,6	157,2	7	8	4,28	1040	**	0,0015
50	109,5	155,7	7	8	4,34	1040	**	0,0011
51	113,2	155,9	7	8	4,01	1040	**	0,0045
52	112,3	156,8	7	8	4,18	1040	**	0,0023
53	112,6	159,7	7	8	4,42	1040	***	0,0008
54	111,9	158,4	7	8	4,37	1040	***	0,001
55	112,0	159,2	7	8	4,43	1040	***	0,0008
56	110,8	158,1	7	8	4,45	1040	***	0,0008
57	109,1	163,0	7	8	5,06	1040	****	< 0,0001
58	112,2	159,3	7	8	4,42	1040	***	0,0008
59	114,7	162,0	7	8	4,45	1040	***	0,0008

Supplementary Table 5: Statistical analyses for Figures 4F and 4G

Data were analyzed with d'Agostino-Pearson and Shapiro-Wilk normality tests.
Time courses were analyzed with 2-way repeated measures ANOVA and post hoc multiple comparisons after ANOVA were done with Holm-Sidak's test.
Comparison of 4 groups were done with 2-way ANOVA (Figs. 3C, 3E, 4E, 4G).
Estimation statistics was done using https://www.estimationstats.com/ which calculated the permutation t-test with 5000 reshuffles (Figs. 3C, 3E, 4E, 4G).
All tests were used as two-tailed.

Figure	Variable	Group	n mice	n slices	Passed normality test	Mean	SEM	95% CI	Statistical analysis	Comparison	DF	Test value	*P* value
**4F**	Normalized slope (% of baseline)	AAV-GFP	5	7					Two-way ANOVA	Interaction	79 ; 948	F = 0,89	0,73
		AAV-GFP/Cre	4	7						Time	79 ; 948	F = 19,12	**< 0,0001**
										Genotype	1 ; 12	F = 0,30	0,59
**4G**	Average potentiation 50-60 min after TBS	AAV-GFP	5	7	Yes[1]	131,0	14,1	96,5 ; 165,5					
		AAV-GFP/Cre	4	7	No	117,9	4,3	107,3 ; 128,5					
		Mean difference between GFP and Cre	8	14		-13,1		-45,9 ; 9,1	Permutation t-test, 5000 reshuffles of GFP and Cre values				0,41

1, Shapiro-Wilk only

4F Post-hoc comparisons

	Normalized slope (% of baseline)		Number of slices		Holm-Sidak's test			
**Time (min)**	**Mean GFP**	**Mean Cre**	**AAV-GFP**	**AAV-GFP/Cre**	**t**	**DF**	**Significance**	**Adj P value**
-20	104,0	104,8	7	7	0,07	960	ns	> 0,9999
-19	101,3	100,3	7	7	0,09	960	ns	> 0,9999
-18	98,0	105,2	7	7	0,65	960	ns	> 0,9999
-17	98,9	105,5	7	7	0,59	960	ns	> 0,9999
-16	99,1	101,3	7	7	0,20	960	ns	> 0,9999
-15	99,5	98,4	7	7	0,10	960	ns	> 0,9999
-14	101,2	99,6	7	7	0,14	960	ns	> 0,9999
-13	97,2	103,2	7	7	0,54	960	ns	> 0,9999
-12	96,2	97,2	7	7	0,09	960	ns	> 0,9999
-11	98,7	100,0	7	7	0,12	960	ns	> 0,9999
-10	100,3	100,2	7	7	0,01	960	ns	> 0,9999
-9	100,7	95,6	7	7	0,46	960	ns	> 0,9999
-8	103,7	101,4	7	7	0,21	960	ns	> 0,9999
-7	97,8	100,0	7	7	0,20	960	ns	> 0,9999
-6	98,7	99,6	7	7	0,08	960	ns	> 0,9999
-5	98,6	101,1	7	7	0,22	960	ns	> 0,9999
-4	102,1	98,5	7	7	0,33	960	ns	> 0,9999
-3	102,8	93,1	7	7	0,87	960	ns	> 0,9999
-2	99,0	95,8	7	7	0,28	960	ns	> 0,9999
-1	102,4	99,2	7	7	0,30	960	ns	> 0,9999

**TBS**	0	118,2	118,1	7	7	0,01	960	ns	> 0,9999
	1	152,4	156,1	7	7	0,33	960	ns	> 0,9999
	2	148,6	150,0	7	7	0,12	960	ns	> 0,9999
	3	147,1	153,2	7	7	0,55	960	ns	> 0,9999
	4	143,5	150,9	7	7	0,66	960	ns	> 0,9999
	5	144,9	146,7	7	7	0,16	960	ns	> 0,9999
	6	140,8	144,5	7	7	0,34	960	ns	> 0,9999
	7	140,2	141,4	7	7	0,11	960	ns	> 0,9999
	8	141,9	140,0	7	7	0,17	960	ns	> 0,9999
	9	139,5	133,0	7	7	0,58	960	ns	> 0,9999
	10	135,3	142,2	7	7	0,62	960	ns	> 0,9999
	11	137,5	133,5	7	7	0,36	960	ns	> 0,9999
	12	135,8	137,9	7	7	0,19	960	ns	> 0,9999
	13	142,0	131,5	7	7	0,94	960	ns	> 0,9999
	14	135,8	131,2	7	7	0,42	960	ns	> 0,9999
	15	138,0	131,1	7	7	0,63	960	ns	> 0,9999
	16	130,9	129,9	7	7	0,09	960	ns	> 0,9999
	17	134,7	126,9	7	7	0,70	960	ns	> 0,9999
	18	130,2	128,0	7	7	0,20	960	ns	> 0,9999
	19	127,8	129,9	7	7	0,19	960	ns	> 0,9999
	20	131,3	128,9	7	7	0,22	960	ns	> 0,9999
	21	128,6	125,9	7	7	0,24	960	ns	> 0,9999
	22	131,6	127,9	7	7	0,34	960	ns	> 0,9999
	23	131,5	123,1	7	7	0,75	960	ns	> 0,9999
	24	131,1	124,0	7	7	0,64	960	ns	> 0,9999
	25	136,2	122,8	7	7	1,21	960	ns	> 0,9999
	26	129,1	123,7	7	7	0,49	960	ns	> 0,9999
	27	134,1	125,1	7	7	0,81	960	ns	> 0,9999
	28	131,4	121,9	7	7	0,85	960	ns	> 0,9999
	29	127,6	121,8	7	7	0,52	960	ns	> 0,9999
	30	132,5	122,0	7	7	0,94	960	ns	> 0,9999
	31	131,7	119,5	7	7	1,09	960	ns	> 0,9999
	32	130,5	121,5	7	7	0,82	960	ns	> 0,9999
	33	133,2	122,8	7	7	0,94	960	ns	> 0,9999
	34	128,2	122,1	7	7	0,56	960	ns	> 0,9999
	35	132,2	125,3	7	7	0,63	960	ns	> 0,9999
	36	131,4	120,4	7	7	0,99	960	ns	> 0,9999
	37	125,9	125,1	7	7	0,07	960	ns	> 0,9999
	38	127,7	123,6	7	7	0,36	960	ns	> 0,9999
	39	129,8	118,2	7	7	1,04	960	ns	> 0,9999
	40	129,3	118,7	7	7	0,95	960	ns	> 0,9999
	41	126,9	122,5	7	7	0,40	960	ns	> 0,9999
	42	125,5	113,8	7	7	1,05	960	ns	> 0,9999
	43	127,0	121,1	7	7	0,53	960	ns	> 0,9999
	44	129,6	118,8	7	7	0,97	960	ns	> 0,9999
	45	127,4	119,8	7	7	0,68	960	ns	> 0,9999

46	129,7	119,1	7	7	0,96	960	ns	> 0,9999
47	128,6	117,9	7	7	0,96	960	ns	> 0,9999
48	130,5	117,4	7	7	1,18	960	ns	> 0,9999
49	128,0	118,5	7	7	0,85	960	ns	> 0,9999
50	128,8	120,4	7	7	0,76	960	ns	> 0,9999
51	129,7	119,6	7	7	0,91	960	ns	> 0,9999
52	134,5	118,4	7	7	1,45	960	ns	> 0,9999
53	128,1	116,6	7	7	1,04	960	ns	> 0,9999
54	133,4	120,4	7	7	1,18	960	ns	> 0,9999
55	134,9	115,2	7	7	1,78	960	ns	0,9982
56	131,7	119,3	7	7	1,12	960	ns	> 0,9999
57	127,0	115,7	7	7	1,02	960	ns	> 0,9999
58	132,3	118,2	7	7	1,27	960	ns	> 0,9999
59	133,0	114,6	7	7	1,66	960	ns	0,9997

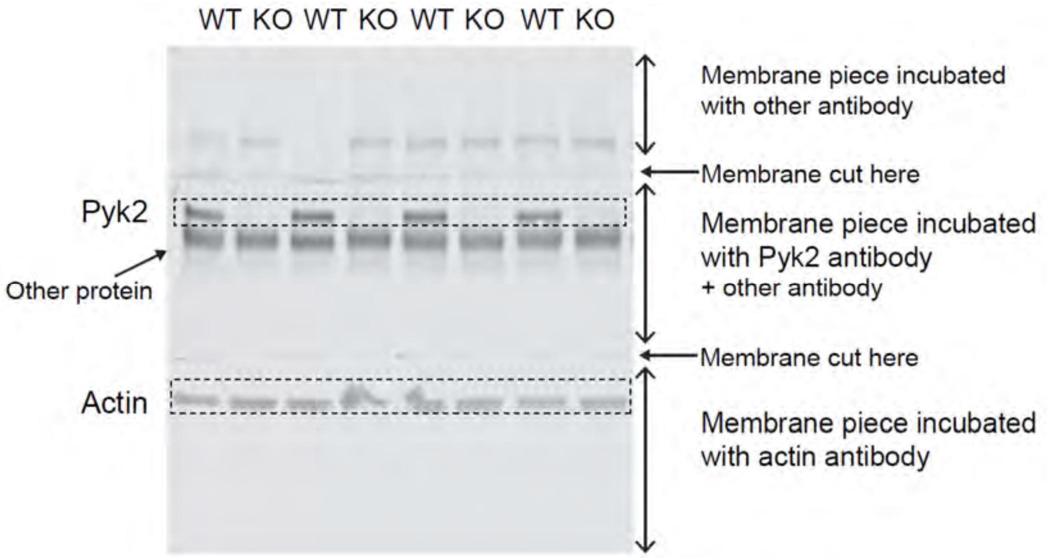

Supplementary Figure 1.

Full length blot used to crop Pyk2 and actin bands shown in Fig. 3A (indicated here by dashed boxes). Hippocampi from wild type (WT) and Pyk2 KO mice, contralateral to those used for electrophysiology, were homogenized and processed as described in the Methods. Proteins were separated by SDS PAGE and transferred to nitrocellulose. The membrane was cut in 3 pieces as indicated by arrows. The 3 parts were incubated with different antibodies. The middle piece was incubated with antibody for Pyk2 and another antibody. The bottom piece was incubated with an actin antibody. After incubation with anti-rabbit IgG DyLight™ 680 conjugated antibodies immunoreactive bands were detected by Odyssey infrared imaging apparatus. See Methods for details.
